# Substituent effect in determining the total structure of an all-alkynyl-protected Ag_98_ nanocluster for methanol tolerant oxygen reduction reaction†

**DOI:** 10.1039/d4sc04318a

**Published:** 2024-10-09

**Authors:** Xiaoqin Cui, Xuehuan Zhang, Ting Li, Sheng Zhu, Gaoyi Han, Huan Li

**Affiliations:** a Institute of Crystalline Materials, Shanxi University Taiyuan 030006 Shanxi China 59584340@sxu.edu.cn; b Institute of Molecular Science, Shanxi University Taiyuan 030006 Shanxi China shengzhu@sxu.edu.cn

## Abstract

Metal nanoclusters (NCs) with atomically precise structures are desirable models for truly understanding their structure–property relationship. This study reports the synthesis and structural anatomy of a Ag_98_ NC protected solely by an alkynyl ligand, 2-(trifluoromethyl)phenylacetylene (2-CF_3_PhC≡CH), which features a –CF_3_ substituent at the *ortho* position (*ortho*-CF_3_). 2-CF_3_PhC≡CH ligands are so exquisitely arranged on the surface of Ag_98_ that the steric hindrance caused by *ortho*-CF_3_ is minimized but its function as a hydrogen-bond (H-bond) acceptor (H⋯F) is maximized. Such a rule also applies to inter-cluster interactions which define the stacking sequence of Ag_98_ NCs. When supported on carbon black, Ag_98_ NCs demonstrate desirable oxygen reduction activity with robust long-term durability and excellent methanol tolerance, outperforming the commercial Pt/C catalyst.

## Abstract 2

A sterically demanding *ortho*-CF_3_ substituent defines not only the arrangement of ligands on a Ag_98_(2-CF_3_PhC≡C)_48_Cl_4_ nanocluster (Ag_98_) but also the inter-cluster interactions. Ag_98_ shows excellent methanol tolerance in the oxygen reduction reaction.

## Graphical abstract

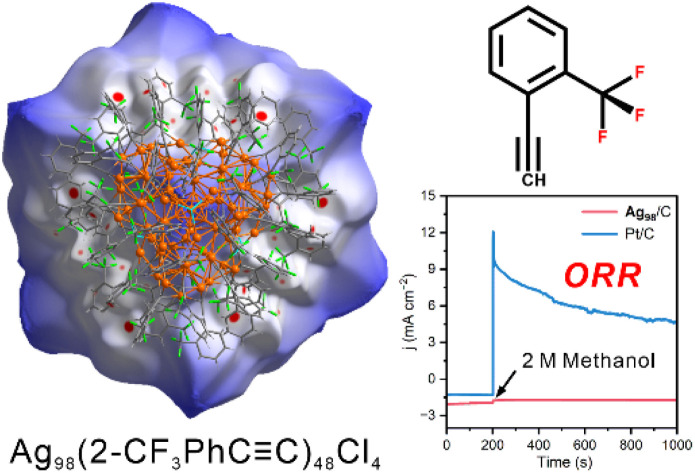

## Introduction

Atomically precise metal nanoclusters (NCs) featuring a small metal core and peripheral ligands are desirable models for understanding their physicochemical properties.^1–5^ To access this type of NC, the choice of ligands is vital. Different ligands have distinct bonding modes, resulting in diverse structures of NCs.^6–9^ The ligands confine the metal core *via* metal–ligand bonding, guarantee the NCs' solubility in solvents, and direct their crystallization behavior through ligand–ligand interactions, not to mention their synergistic effects in optical and catalytic applications.^10–12^ Therefore, understanding the role of ligands has aroused keen interest in recent years.^13–15^ Up to now, a variety of organic ligands such as thiols,^16–20^ phosphines,^21,22^ alkynes^23–26^ and carbenes^27–29^ have been successfully applied in the synthesis of coinage metal NCs (Au, Ag, Cu).^30^

Alternatively, a metal NC can also be regarded as a certain number of ligands being anchored densely on an extremely small metal nanoparticle (usually less than 2 nm). In such narrow space, steric hindrance is pronounced, especially considering the ligands often have a bulky benzene ring as an integral part. The ligands are also restricted by their specific bonding mode. For example, PPh_3_ usually coordinates with one metal atom, while thiolate tends to form a staple bonding motif.^31^ Besides, as single crystals, ligands must also strictly comply with crystallographic rules. Constrained by these conditions, ligands tend to exhibit highly regular and symmetric patterns, which in turn shape the outermost layer of metal atoms.^32,33^ Thus, studying the distribution and coordination patterns of ligands will provide key information for understanding how the ligands shape the NC.^34^

Previously, the complete structure of alkynyl-protected Ag NCs (*e.g.*, Ag_32_,^35^ Ag_42_,^36^ Ag_48_,^37^ Ag_51_,^38^ Ag_74_,^39,40^ Ag_112_ (ref. 41) and Ag_148_ (ref. 42)) has been determined.^43^ They mostly employed alkynyl ligands either without any substituent or with substituents at symmetric positions, such as phenylacetylene,^36,39,40^ 3,5-bis(trifluoromethyl) phenylacetylene,^23,35,41^*tert*-butylacetylene^25,37,44^*etc.* The potential role of an asymmetrically arranged substituent group in determining the crystal structure of metal NCs has yet to be truly unraveled. In this work, a Ag_98_(2-CF_3_PhC≡C)_48_Cl_4_ (denoted as Ag_98_) NC protected by 2-(trifluoromethyl)phenylacetylene (*o*-TPA) is synthesized and characterized. *O*-TPA has one sterically demanding –CF_3_ substituent at the *ortho* position (*ortho*-CF_3_). It causes the *o*-TPA ligand to lose its *C*_2_ symmetry in comparison with phenylacetylene. This can be used as an indicator of ligand orientation.

Hence, Ag_98_ where 48 alkynyl ligands are pinned on a 1.5 nm Ag nanoparticle is a desirable model for studying the substituent effect in the following aspects. Firstly, how does the bulky *o*-TPA adapt to the narrow surface when a sterically demanding *ortho*-CF_3_ is present? Secondly, how does the arrangement of surface ligands shape the Ag–ligand interface? Thirdly, will *ortho*-CF_3_ influence the inter-cluster interaction and accordingly the whole crystal structure of Ag_98_? Aiming to resolve these puzzles, a comprehensive structural analysis of Ag_98_ is implemented. The decisive role of *ortho*-CF_3_ in shaping the crystal structure is revealed. When supported on activated carbon, Ag_98_ exhibits excellent oxygen reduction reaction (ORR) activity with superior durability and methanol tolerance compared with a commercial Pt/C catalyst.

## Experimental

### Materials and methods

All the chemicals were of analytical grade, obtained from commercial sources and used as received. Bis(diphenylphosphino)propane (Dppp) and 2-(trifluoromethyl)phenylacetylene (2-CF_3_PhC≡CH) were purchased from Adamas Reagent Co., Ltd. Silver nitrate (AgNO_3_, 99.8%) and sodium borohydride (NaBH_4_, 98%) were from Energy Chemical (Shanghai, China). Dichloromethane and the other reagents employed were purchased from Sinopharm Chemical Reagent Co., Ltd (Shanghai, China).

### Synthesis of Ag_98_(2-CF_3_PhC≡C)_48_Cl_4_ nanoclusters

AgNO_3_ (17 mg, 0.1 mmol) was dissolved in 1 mL ethanol, then a 2 mL CH_2_Cl_2_ solution containing Dppp (1,3-bis(diphenylphosphino)propane) (21 mg, 0.05 mmol) and 2-CF_3_PhC≡CH (14 μL, 0.1 mmol) was added under vigorous stirring. A freshly prepared solution of NaBH_4_ (0.1 mmol in 1 mL ethanol) was added dropwise after that. The reaction was aged for 30 h under ambient conditions, during which the color further changed from light yellow to dark brown red. The isolated precipitate was dissolved in CH_2_Cl_2_ (2 mL) and centrifuged for 10 min at 12 000 rpm. The supernatant solution was layered with *n*-hexane and diethyl ether (volume ratio, 1 : 1) for diffusion. Black block crystals were obtained after one week with a yield of 24% (based on Ag).

### Electrochemical test

The ORR performance of the catalysts was tested by cyclic voltammetry (CV) and linear sweep voltammetry (LSV) on a CHI 760E electrochemical workstation with a typical three-electrode system, in which a rotating disk electrode (RDE, *d* = 3.0 mm) loaded with catalysts was used as the working electrode, Ag/AgCl (saturated KCl) as the reference electrode and Pt wire as the auxiliary electrode. The CV and LSV curves were recorded in O_2_-saturated 0.1 M KOH electrolyte. All potentials were converted to the reversible hydrogen electrode (RHE) scale according to the formula:*E*_RHE_ = *E*_Ag/AgCl_ + 0.0592 pH + 0.198 V

### Physical measurements

UV-vis absorption spectra were recorded on a TU-1950 UV-vis spectrophotometer with samples being dispersed in CH_2_Cl_2_. FT-IR spectra were collected on a Nicolet iS5 with samples prepared as KBr pellets. X-ray Photoelectron Spectroscopy (XPS) for Ag, C, F, and Cl was carried out using a monochromatic Al Kα (1486.69 eV) X-ray source operated on a Thermo Fisher Scientific K-Alpha, and the spectra were calibrated using the C 1s peak at 284.8 eV. Thermogravimetric analysis (TGA) was performed on a Setaram Labsys Evo TG-DSC/DTA analyzer in a N_2_ atmosphere with a heating rate of 10 °C min^−1^ from 50 °C to 800 °C. The powder X-ray diffraction (XRD) analysis of the obtained crystal samples was carried out using a Bruker D2 PHASER diffractometer with Cu Kα radiation ranging from 5° to 90°.

### Crystallography

The crystallographic data of Ag_98_ was obtained on an Agilent Technologies SuperNova Single Crystal Diffractometer using Cu Kα radiation (*λ* = 1.54184 Å) at 150 K. Absorption corrections were applied by using the program CrysAlis^45^ (multi-scan). The structure was solved and refined using full-matrix least-squares based on *F*^2^ with program SHELXT and SHELXL within OLEX2.^46^ All the non-hydrogen atoms were refined anisotropically and H atoms isotropically; all hydrogen atoms were generated geometrically and constrained to ride on their parent atoms. For the –CF_3_ group, RIGU, SIMU, DFIX and DANG constraints are often applied due to disorder. The bond lengths of C–F were set to ∼1.35 ± 0.01 Å. The bond lengths of C–C were set to ∼1.54 ± 0.01 Å. The bond lengths of C≡C were set to ∼1.20 ± 0.01 Å. Meanwhile the distances between the 1,3-atoms of C⋯F and F⋯F were set to ∼2.5 ± 0.01 Å and 2.2 ± 0.01 Å. Some aromatic rings were treated by using rigid constraints (AFIX 66).

## Results and discussion

### Crystal structure from single crystal X-ray diffraction

**Fig. 1:**
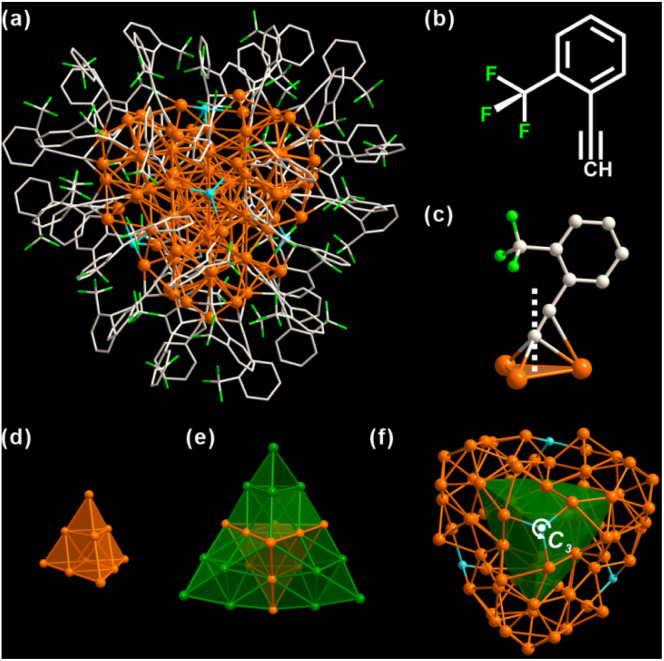
(a) Overall structure of Ag_98_(2-CF_3_PhC≡C)_48_Cl_4_ (Ag_98_). (b) Structure of 2-CF_3_PhC≡CH (*o*-TPA). (c) Bonding motif of *o*-TPAs with Ag atoms. The white dotted line is the normal line from the alkynyl C atom to the Ag_3_ plane. (d) Inner Ag_10_ tetrahedron. (e) Ag_10_@Ag_16_ shells. (f) Ag_10_@Ag_16_@Ag_72_ core viewed along the *C*_3_-axis. Ag_16_ is represented by a green polyhedron. Color labels: orange and green, Ag; grey, C; blue, Cl; fluorescent green, F; white, H.

As shown in Fig. 1, Ag_98_ is a 1.5 nm Ag metal core protected by 48 *o*-TPAs and 4 Cl ligands (Fig. S2†). It crystallizes in a trigonal lattice, *R*3̄ space group (Fig. S3†). All 48 *o*-TPAs employ the μ_3_-bridging mode (Fig. 1c and S4†) *via* σ-type or π-type bonds. Notably, *ortho*-CF_3_ causes the benzene rings to tilt in the opposite direction from the CF_3_ group. This is true for almost every *o*-TPA ligand, whose tilt angles range from 14° to 43° (Table S1†). Apparently, *ortho*-CF_3_ introduces steric hindrance into the local coordination environment. The metal core consists of 98 silver atoms that are arranged into a Ag_10_@Ag_16_@Ag_72_ three-shell architecture. The innermost core is a Ag_10_ tetrahedron. It can be viewed as a unit cut from a face-centered cubic (FCC) silver (Fig. 1d). The average Ag–Ag bond distance of the Ag_10_ shell is 2.884 Å (Fig. S5†), close to that in bulk Ag (2.889 Å).^47^ This shows the metallic nature of Ag_98_. Each of the four tetrahedron facets is extended by another Ag_10_ tetrahedron. In this fashion, Ag_10_@Ag_16_ appears as a star-shaped polyhedron (Fig. 1e). The outermost Ag_72_ shell is composed of four bowl-like Ag_18_ units. Each one caps a convex tetrahedron of the star polyhedron (Fig. S6†). Four μ_3_-Cl moieties are tetrahedrally arranged, through one of them passes the 3-fold rotation axis (Fig. 1f). Considering there is no Cl element in the metal precursor or ligands, it likely results from the solvent, CH_2_Cl_2_.

**Fig. 2:**
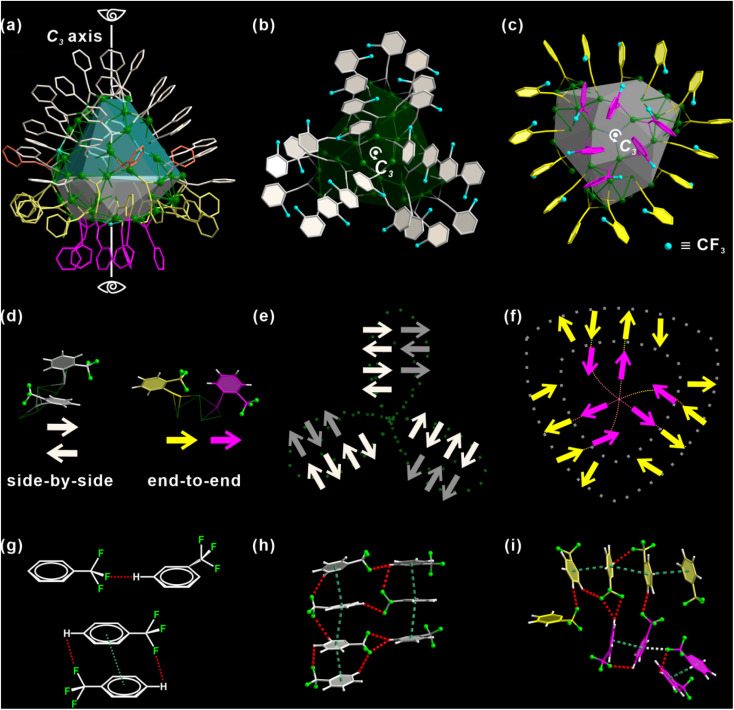
(a) Distribution of the ligands on Ag_98_. They are separated into groups by different colors. “Three-leaf clover” (b) and “shield-like” (c) ligand ensembles of Ag_98_. (d) “Side-by-side” and “end-to-end” position relations between two neighboring *o*-TPA ligands. Simplified schematic illustration of the *o*-TPA ligand of “three-leaf clover” (e) and “shield-like” (f) structures. An arrow represents an *o*-TPA ligand. It approximately points from the center of the benzene ring to the carbon atom of *ortho*-CF_3_. (g–i) The intra-cluster C–F⋯H (red dashed lines), π–π (green dashed lines) and C–F⋯π (white dashed line) interactions. Color labels: green, Ag; gray, orange, yellow and purple, C; fluorescent green, F; blue, Cl; white, H.

There is a total of 48 alkynyl ligands, *o*-TPAs, on Ag_98_. For ease of discussion, the metal core is divided into two areas, colored in green and gray respectively (Fig. 2a). *O*-TPAs are accordingly divided into four groups: 24 white, 15 yellow, 6 purple, and 3 orange *o*-TPAs. 24 white *o*-TPAs form a “three-leaf clover” structure (Fig. 2b). Each “leaflet” includes eight *o*-TPAs that can be further divided into two equal parallel subgroups. The bottom view in Fig. 2c includes 21 *o*-TPAs. They form a “shield-like” shape.

Six purple *o*-TPAs are arranged into a triangle that is surrounded by the outer 15 yellow *o*-TPAs. For convenience, each *o*-TPA is represented by an arrow, which approximately points from the center of the benzene ring to the carbon atom of *ortho*-CF_3_, as explained in Fig. 2d. As such, “three-leaf clover” and “shield” ligand ensembles are simplified as the patterns in Fig. 2e and f. It is shown that for two adjacent *o*-TPA ligands, they have two positional relationships: “side-by-side” and “end-to-end” (Fig. 2d). For any two neighboring “side-by-side” *o*-TPAs of either “three-leaf clover” or “shield” ligand ensembles, their arrows point in opposite directions so their *ortho*-CF_3_ moieties are away from each other. Their benzene rings are arranged nearly parallelly. This not only minimizes the steric hindrance between *o*-TPAs but also lets them fully interact *via* C–F⋯H and π–π interactions, as illustrated in Fig. 2g. For the “end-to-end” *o*-TPA ligand pair, their arrows point in the same direction. This avoids direct contact between the two *ortho*-CF_3_ and guarantees the formation of C–F⋯H hydrogen bonds (H-bond) (Fig. 2g). It is conceivable that if one of the two adjacent *o*-TPAs points to the opposite direction, two *ortho*-CF_3_ would contact directly. Fig. 2h and i provide the details of multiple C–F⋯H H-bonds (red dashed lines). Basically, each *ortho*-CF_3_ group functions as a hydrogen bond acceptor. The distances of H-bonds are in the range of 2.6 to 3.0 Å. In other words, *o*-TPAs are arranged in such an exquisite manner that the capability of *ortho*-CF_3_ as an H-bond acceptor is maximized but the steric effect caused by it is minimized. As shown in Fig. S7,† the steric effect is analyzed using the web tool SambVca 2.^47^ The closely arranged dark red strips indicate prominent steric hindrance caused by the rigid benzene rings. The areas enclosed by green dashed boxes result from *ortho*-CF_3_. Their orange color indicates a significant steric effect caused by *ortho*-CF_3_, although it is slightly less than that of the benzene ring.

The distribution of *o*-TPAs and the surface Ag atoms are interdependent. Each “leaflet” of the “three-leaf clover” structure includes 19 edge-sharing Ag_3_ triangles (Fig. S8a†) capped by eight *o*-TPAs. Each *o*-TPA bonds with a Ag_3_ triangle around the periphery of the “leaflet”. In the purple part, six Ag_3_ triangles are connected by corner-sharing to form a large Ag_6_ triangle (Fig. S8b†). A Cl atom is at the center through which passes the *C*_3_ rotation axis. Thirty-six Ag atoms in the yellow part form six linked pentagonal pyramids. Two apexes of neighboring pyramids are located either above or below the pentagon base (Fig. S8c and S8f†). All *o*-TPA are in the μ_3_-bridging mode (Fig. S8†).

**Fig. 3:**
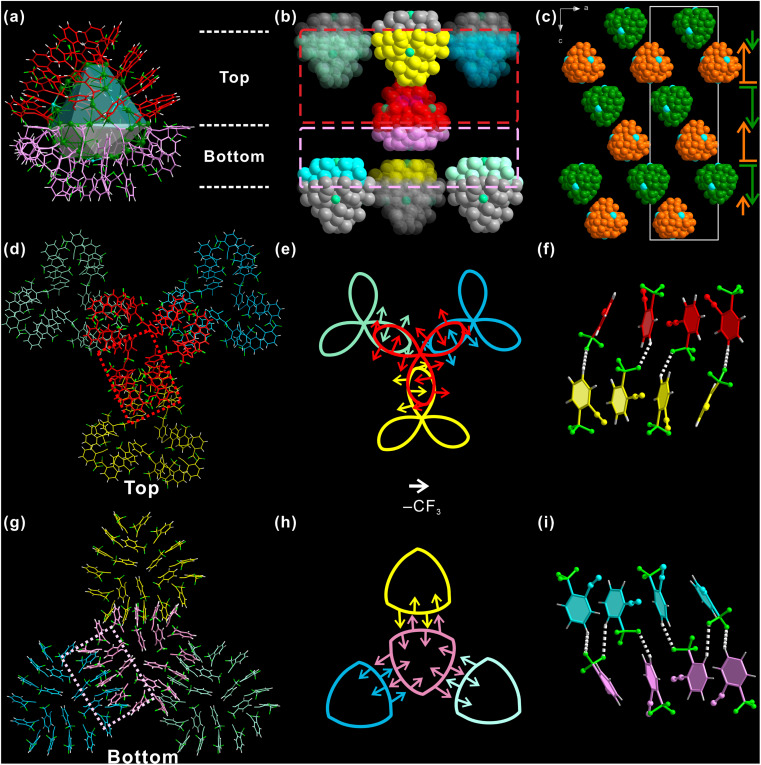
(a) Forty-eight 2-CF_3_PhC≡CH (*o*-TPA) ligands colored in red and pink on Ag_98_. (b) One centered Ag_98_ (red and pink) with its six neighboring Ag_98_ NCs. (c) Orientations of Ag_98_ NCs. Different orientations are shown in different colors (orange or green). Cross-section views of “three-leaf clover” ligands (d) and “shield-like” ligands (g) of one Ag_98_ interacting with the same parts of another three Ag_98_. (e) and (h) are simplified schematic representations of the patterns (d) and (g), respectively. An arrow is an *o*-TPA, it approximately points from the center of the benzene ring to the carbon of the *ortho*-CF_3_ substituent. (f) and (i) are the details of bonding of (e) and (h). The color code is the same as that in (e) and (h).

Not only the individual Ag_98_, but also the inter-cluster structure is significantly affected by *ortho*-CF_3_. For ease of discussion, Ag_98_ is also divided into two parts: the “three-leaf clover” top part (red, Fig. 3a) and a “shield-like” bottom part (pink). Each such Ag_98_ has six nearest neighbor Ag_98_ NCs, and each side has three. As shown in Fig. 3b, the top part of the center Ag_98_ is in close proximity to the same parts of another three Ag_98_ NCs (red dashed box area in Fig. 3b). The situation is similar for the bottom part. Such an arrangement necessitates the opposite orientations of Ag_98_ NCs of adjacent layers. Such a situation is illustrated in the view along the *b*-axis (Fig. 3c). Correspondingly, the inter-cluster ligand interactions are either “leaf-to-leaf” or “shield-to-shield” (Fig. 3d and g). A perpendicular view of the involved top area as framed by a red dashed rectangle (Fig. 3b) is shown in Fig. 3d. One “leaflet” overlaps with a “leaflet” of another Ag_98_. In this way, the “three-leaf clover” pattern forms interactions with three neighboring Ag_98_ NCs.

To better understand such a pattern, “three-leaf clover” is represented by a Kiepert curve that also has a *C*_3_ rotation symmetry (Fig. 3e). Each arrow of it stands for one *o*-TPA ligand and it points from the center of the benzene ring to *ortho*-CF_3_, the same as discussed above. It is found that although these *o*-TPAs are from different Ag_98_ NCs, they still follow the rule observed on an individual NC. That is “side-by-side” *o*-TPAs have their *ortho*-CF_3_ facing the opposite directions and “end-to-end” *o*-TPAs have their *ortho*-CF_3_ pointing in the same direction. As shown in Fig. 3f, a red and a yellow *o*-TPA from respective Ag_98_ NCs are arranged end-to-end, forming a C–F⋯H H-bond. The neighboring pairs of *o*-TPA have similar interactions but face the opposite direction. Similarly, the ligands in the “shield-like” part are simplified to the Reuleaux triangle. In Fig. 3h, the rule observed on the Kiepert curve also applies to the Reuleaux triangle (Fig. 3i). Thus, the orientation and distribution of *ortho*-CF_3_ is not only critical for determining the ligand arrangement on an individual Ag_98_, but is also decisive in shaping the inter-cluster structure.

**Fig. 4:**
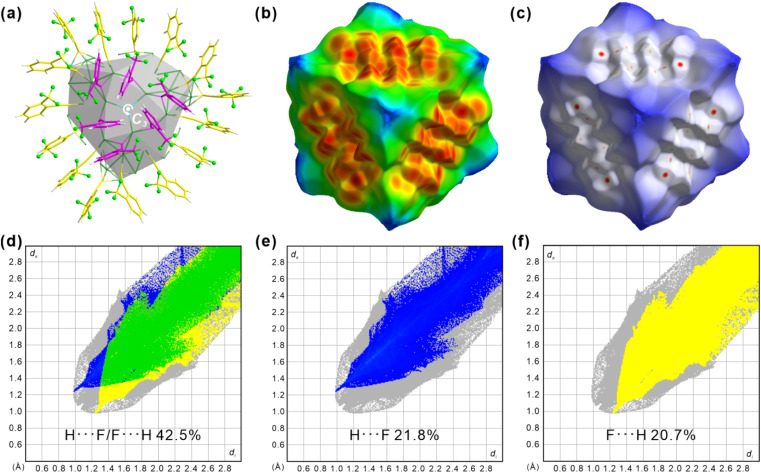
(a) “Shield-like” part of Ag_98_ viewed along the *c*-axis. Hirshfeld surface of the structure in (a) as mapped by *d*_e_ (b) and *d*_norm_ (c) surfaces. Fingerprint plots (d) H⋯F/F⋯H, (e) H⋯ and (f) F⋯H contacts. The full fingerprint appears beneath each decomposed plot as a grey shadow.

The Hirshfeld surface, which defines an outer surface of electron density, encodes information about intermolecular interactions.^48,49^ The analytical results for the “shield-like” part are shown in Fig. 4. The external distances from the Hirshfeld surfaces to the nearest nucleus, *d*_e_, are shown in Fig. 4b. Three red areas which indicate short-distance interactions are trigonally distributed around the center. This results from the proximity of another three trigonally distributed Ag_98_ NCs, as described in the crystallographic results (Fig. 3 and S9†). The normalized distance (*d*_norm_) highlights the contacts shorter than the van der Waals radius as red spots (Fig. 4c).

Three groups of red spots have nearly the same numbers and shapes, corresponding to the same bond numbers and distances. A decomposition analysis in Fig. S10† proves that the H⋯F/F⋯H H-bond dominates the short interactions, although F⋯F and H⋯H are also present. Furthermore, the fingerprint plot in Fig. 4f shows two spikes which have the characteristic shape for H-bonding (upper one for a H-bond donor and lower one for an acceptor). Ag_98_ has its one *o*-TPA functioning as an acceptor (F atom) and its neighboring *o*-TPA as a donor (H of benzene) (Fig. S11†). In all, H⋯F/F⋯H H-bonding contributes 42.5% to the Hirshfeld surface (Fig. S12 and S13†), much higher than other non-H⋯H contacts. In other words, C–F⋯H H-bonds dominate the inter-cluster interactions and direct the packing sequence of Ag_98_. This further highlights the importance of the *ortho*-CF_3_ substituent as an H-bond acceptor.

Ag_98_ was characterized by different techniques. The disappearance of the stretching mode of *ν*(≡C–H) at 3300 cm^−1^ in the FT-IR spectrum of Ag_98_ indicates the coordination of *o*-TPA with Ag (Fig. S14†). Upon addition of NaBH_4_, a new absorption peak in the UV-vis spectrum appeared, which gradually red-shifted from 398 to 432 nm, and finally to 522 nm during aging (Fig. S15 and S16†). It is characteristic of Ag_98_. X-ray photoelectron spectroscopy (XPS) of Ag_98_ reveals all expected elements of Ag_98_ (Fig. S18 and S19†).^50^ The Ag 3d_5/2_ signals can be deconvoluted into signals at 369.1 eV (3d_5/2_, Ag^0^) and 366.9 eV (3d_5/2_, Ag^+^). Also, the thermogravimetric analysis (TGA) curve proves that Ag_98_ remains stable up to 180 °C (Fig. S21†).

**Fig. 5:**
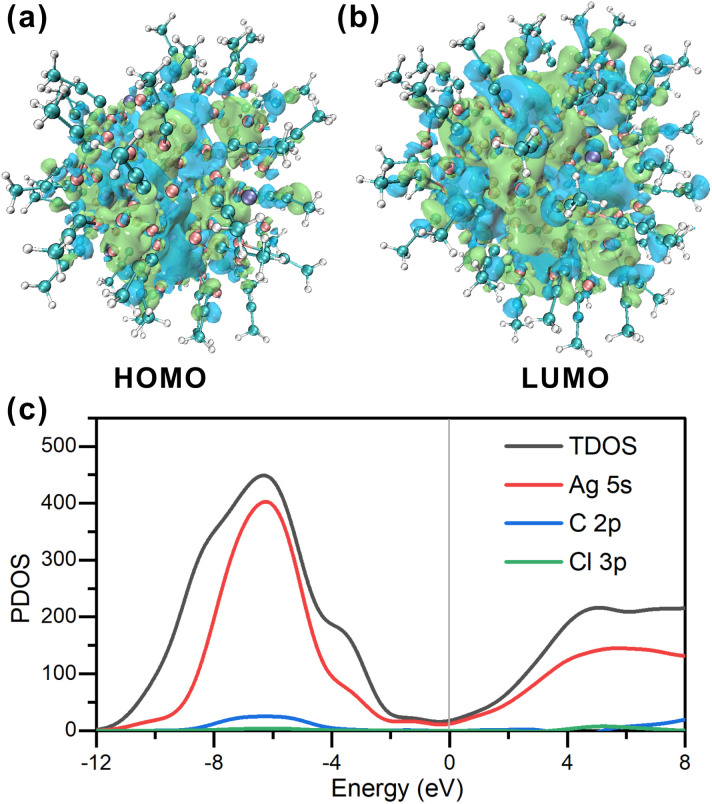
(a and b) Frontier orbitals of Ag_98_. (c) PDOS of Ag_98_. The HOMO levels are positioned at the zero-energy level.

To gain further insights, the electronic structure of Ag_98_ was examined using density functional theory (DFT) calculations. To simplify the computations, benzene rings were replaced with methyl groups. The neutral singlet state of the Ag cluster displays a unique and stable electronic configuration. Fig. 5 accurately illustrates the spatial distribution of the highest occupied molecular orbital (HOMO) and the lowest unoccupied molecular orbital (LUMO). The HOMO orbitals, being the least stable electron residence, indicate areas on the Ag cluster where electrons are most likely to detach, particularly around the –CH_3_ groups and the –Ag–C≡C motifs. Conversely, the LUMO orbitals highlight the potential sites for the cluster to accept additional electrons, which are centered around the Ag core at their lowest energy state, thus predicting the cluster's potential electron affinity. Specifically, in the neutral singlet state, the HOMO has an energy of −2.709 eV, while for the LUMO it is −2.365 eV, yielding a HOMO–LUMO gap of 0.344 eV. Analysis of the total density of states (TDOS) in Fig. 5c indicates that the Ag cluster exhibits metallic characteristics, with an even distribution of state density near the HOMO orbital energy level. This corresponds to an sp-like band, suggesting that the Ag cluster possesses electron delocalization properties. Additionally, there are prominent peaks at −6.2 eV and 4.9 eV, indicating the presence of pseudo energy gaps (1.5 eV). This further suggests that the Ag cluster exhibits covalent bonding character. The primary contributions to the DOS near the Fermi level come from Ag 5s orbital electrons. This indicates that the potential sites for accepting electrons are around the Ag atoms, which should facilitate its electrocatalytic applications.

### Electrocatalytic ORR with Ag_98_

**Fig. 6:**
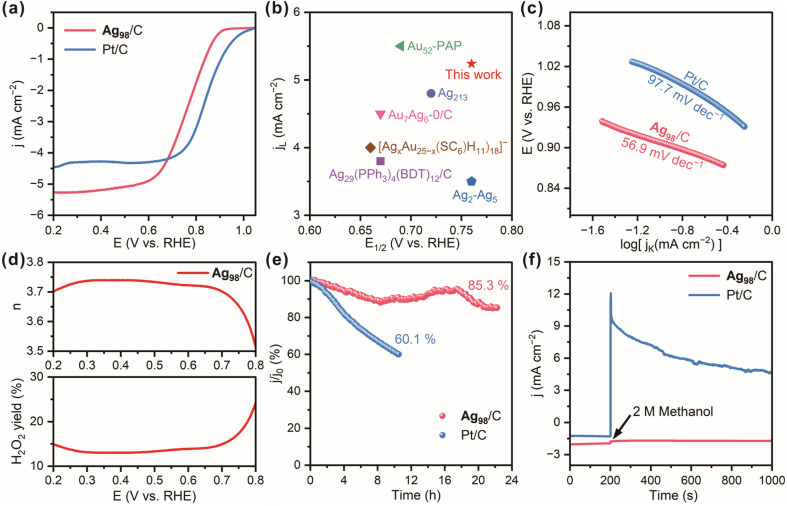
(a) ORR LSV curves of Ag_98_/C and Pt/C in an O_2_-saturated 0.1 M KOH solution at a scan rate of 10 mV s^−1^ and a rotation speed of 1600 rpm. (b) Comparison of the *E*_1/2_ and *j*_L_ values between Ag_98_/C and the reported cluster catalysts. (c) The Tafel slopes of Ag_98_/C and Pt/C catalysts. (d) Electron transfer numbers (*n*) and H_2_O_2_ yield of Ag_98_/C. (e) Chronoamperometric curves for the ORR of Ag_98_/C and Pt/C catalysts. (f) The methanol-tolerance performances of Ag_98_/C and Pt/C catalysts.

Thus, the ORR catalytic performance of the Ag_98_/C material was evaluated on a glassy carbon electrode in a three-electrode system with a 0.1 mol L^−1^ KOH electrolyte. A carbon rod and an Ag/AgCl electrode served as the counter electrode and reference electrode, respectively. The tunable d-orbital electronic structure of the transition metal silver allows for an optimal adsorption energy for oxygen molecules, which could effectively reduce the activation energy barrier of the ORR. Compared to the bulk materials, Ag NCs possess a high specific surface area and more active sites. These effects enhance the electron transfer efficiency between Ag NCs and reactants, thereby modulating both the reaction pathway and activity of the ORR.^51^ From the linear sweep voltammetry (LSV) curves in Fig. 6a, it can be observed that the Ag_98_/C exhibits desirable ORR performance with a high half-wave potential (*E*_1/2_) of 0.76 V and a large limiting current density (*J*_L_) of −5.2 mA cm^−2^, close to that of the Pt/C catalyst (*E*_1/2_: 0.84 V, *J*_L_: −4.5 mA cm^−2^). Notably, the oxygen reduction potential and *E*_1/2_ of Ag_98_/C significantly exceed those of pure carbon black (Fig. S23†), revealing the desirable ORR electrocatalytic activity of Ag_98_. Moreover, the *E*_1/2_ and *j*_L_ values of Ag_98_/C are even superior to those of most recently reported similar electrocatalysts (Fig. 6b and Table S2†).^30,52–55^ On the other hand, Ag_98_/C samples were also treated by a fast thermal treatment method to remove the ligands without sintering them.^56^ They displayed a slightly reduced *E*_1/2_ value (0.66 V), suggesting the surface ligands had a minor promoting effect on the ORR performance, which could be ascribed to the electronic effect caused by the bonding with alkynyl ligands (Fig. S24†). Also, the Tafel slope of the Ag_98_/C is calculated to be 56.9 mV dec^−1^, lower than that of Pt/C (97.7 mV dec^−1^, Fig. 6c), demonstrating its facilitated kinetics and higher efficiency in the catalytic process of ORR. As shown in Fig. 6d, the H_2_O_2_ yield and electron transfer number (*n*) calculated from the LSV curves measured on a rotating ring-disk electrode (RRDE) are 14.5% and 3.7, respectively, evaluating the selectivity of Ag_98_/C for O_2_ reduction through near 4e^−^ transfer steps. Additionally, Ag_98_/C achieves prominent durability with high current retention of 85.3% after continuous operation for 23 h, whereas Pt/C retains only 60.1% after 10 h of cycling (Fig. 6e). This may be attributed to the high dispersion of silver nanoclusters over the conductive carbon substrate and strong interactions, which could prevent nanoclusters from easy abscission during continuous stability testing. Also, the chronoamperometric curve of Ag_98_/C in Fig. 6f remains nearly unchanged after methanol injection, while the current for Pt/C significantly declines, revealing the superior methanol tolerance of the Ag_98_ NCs compared to the Pt/C catalyst. This is due to silver having a relatively low catalytic activity compared to methanol, which reduces its corrosion by methanol.^57^ Additionally, the electronic structure of silver allows it to preferentially adsorb oxygen molecules in the ORR, further decreasing the accessibility to methanol. In contrast, platinum is more prone to reacting with methanol, leading to poor methanol tolerance performance.^58^ The steric hindrance of the *ortho*-CF_3_ ligand may also make it difficult for methanol molecules to approach the active sites, thereby further enhancing its methanol tolerance performance.

## Conclusions

This work highlights the unanticipated significance of an *ortho*-CF_3_ substituent of an alkynyl ligand in determining not only the distribution of ligands on an individual Ag nanocluster but also the inter-cluster interactions. The steric hindrance caused by *ortho*-CF_3_ is minimized but its function as an H-bond acceptor is maximized. When supported on carbon black, Ag_98_ nanoclusters exhibit excellent methanol tolerance and robust long-term durability in the oxygen reduction reaction.

## Data availability

Data are available on request from the authors.

## Author contributions

Ting Li and Xiaoqin Cui: synthesis, structural analysis. Huan Li and Xiaoqin Cui: writing of the paper. Ting Li and Xiaoqin Cui: data collection on an X-ray diffractometer and crystal structure refinement. Xuehuan Zhang and Xiaoqin Cui: data testing and analysis of electrocatalytic ORR. Huan Li, Sheng Zhu and Gaoyi Han: conceptualization, funding, and revision of the paper. All authors have discussed the results and contributed to the final manuscript.

## Conflicts of interest

There are no conflicts to declare.

## Supplementary Material

SC-OLF-D4SC04318A-s001

SC-OLF-D4SC04318A-s002
