# Supplementary material for: Substituent effect in determining the total structure of an all-alkynyl-protected Ag98 nanocluster for methanol tolerant oxygen reduction reaction
Source: Chem Sci. 2024 Oct 9;15(43):18161–9. doi: 10.1039/d4sc04318a (PMC11474724; doi:10.1039/d4sc04318a)
Supplement: SC-015-D4SC04318A-s001 [file SC-015-D4SC04318A-s001.pdf]

Supplementary Information (SI) for Chemical Science.
This journal is © The Royal Society of Chemistry 2024

# Supporting Information

## Substituent Effect in Determining the Total Structure of an All-Alkynyl-Protected $Ag_{98}$ Nanocluster for Methanol-Tolerant Oxygen Reduction Reaction

Xiaoqin Cui[a†], Xuehuan Zhang[b†], Ting Li[a†], Sheng Zhu[b*], Gaoyi Han[b] and Huan Li[a*]

a. Institute of Crystalline Materials, Shanxi University, Taiyuan 030006, Shanxi, China; Email: 59584340@sxu.edu.cn (H. L.).

b. Institute of Molecular Science, Shanxi University, Taiyuan 030006, China. Email: shengzhu@sxu.edu.cn (S. Z.).

† These authors contributed equally.

## METHODS

### I. Hirshfeld Surfaces Study

The crystallographic information contained in the cif file was used to perform computational calculations. The Hirshfeld surfaces and their associated 2D fingerprint plots were generated using the CrystalExplorer 21.5 software. The $d_e$, $d_{\text{norm}}$ (normalized contact distance) surface and the breakdown of the 2D fingerprint plots were used to investigate the intermolecular interactions.

### II Density Functional Theory Calculations

All calculations presented in this study were meticulously conducted utilizing the Gaussian 16 software package. The geometry optimization and structural analyses of the investigated molecular systems were

performed at the B3LYP with the Lanl2DZ basis set [1], which is specifically designed for the accurate description of transition metal elements, ensuring that the electronic structures of the studied compounds were accurately captured. Furthermore, to gain deeper insights into the electronic properties of these structures, the Total Density of States (TDOS) was analyzed using the Multiwfn 3.8 (dev) software package [2]. To visualize The HOMO and LUMO were visualized using VMD (Visual Molecular Dynamics) software [3].

## III. Characterization

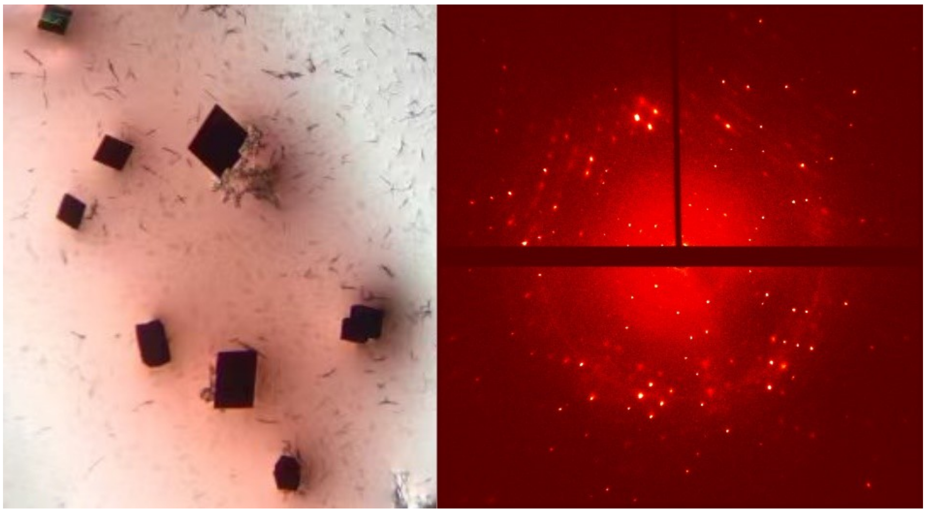

**Figure S1**. Optical microphotograph and X-ray diffraction Laue photograph of $\mathbf{Ag_{98}}$ single crystals (black block).

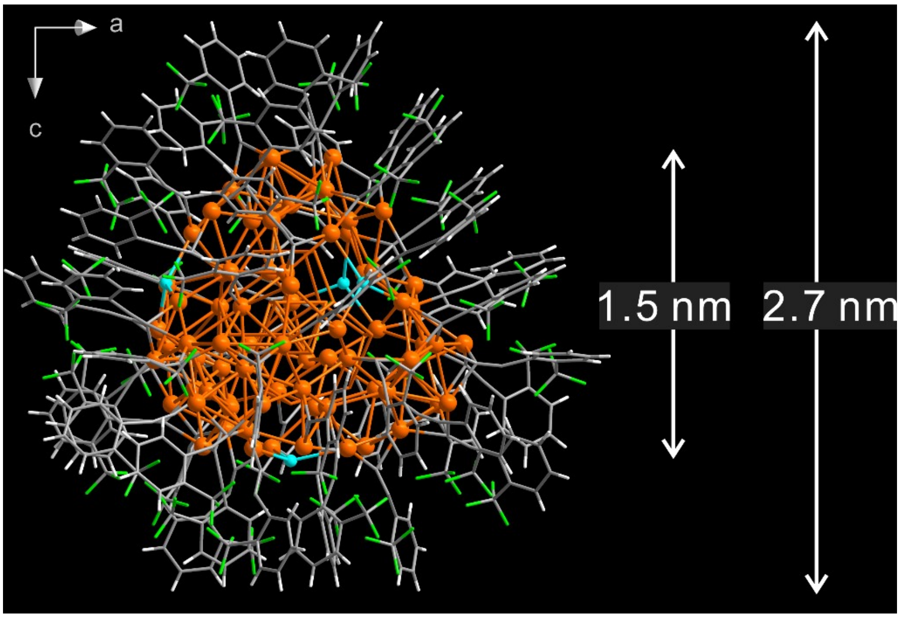


**Figure S2.** The overall size and core size of the $Ag_{98}(2\text{-}CF_3PhC{\equiv}C)_{48}Cl_4$ nanoclusters. Color legend: Ag, orange; C, grey; F, green; Cl, blue; H, white.

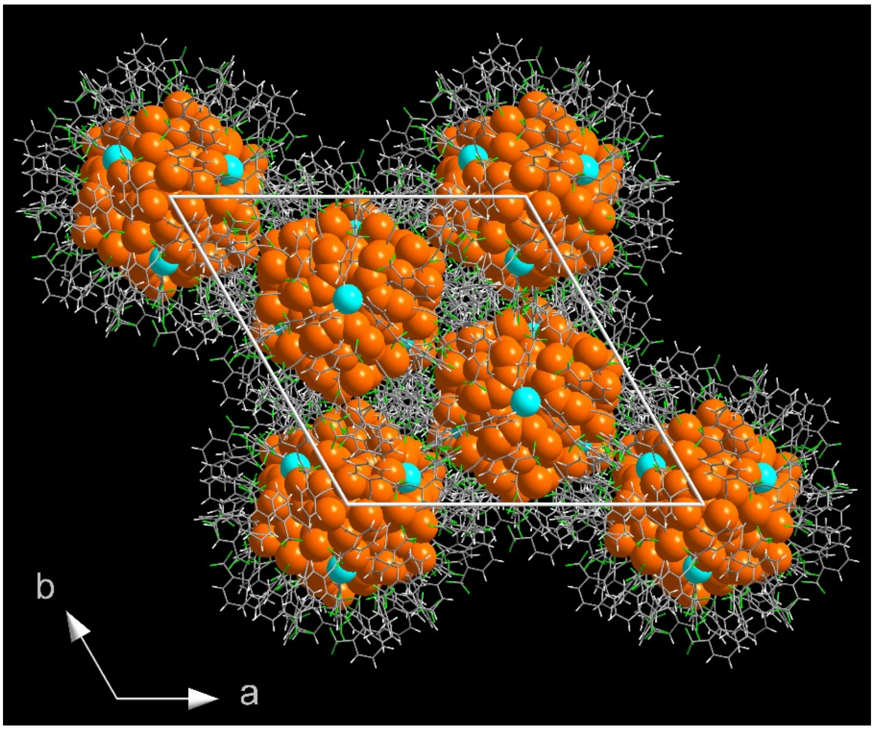


**Figure S3**. Packing of $\mathbf{Ag_{98}}$ in a unit cell. Color legend: Ag, orange; C, grey; F, green; Cl, blue; H, white.

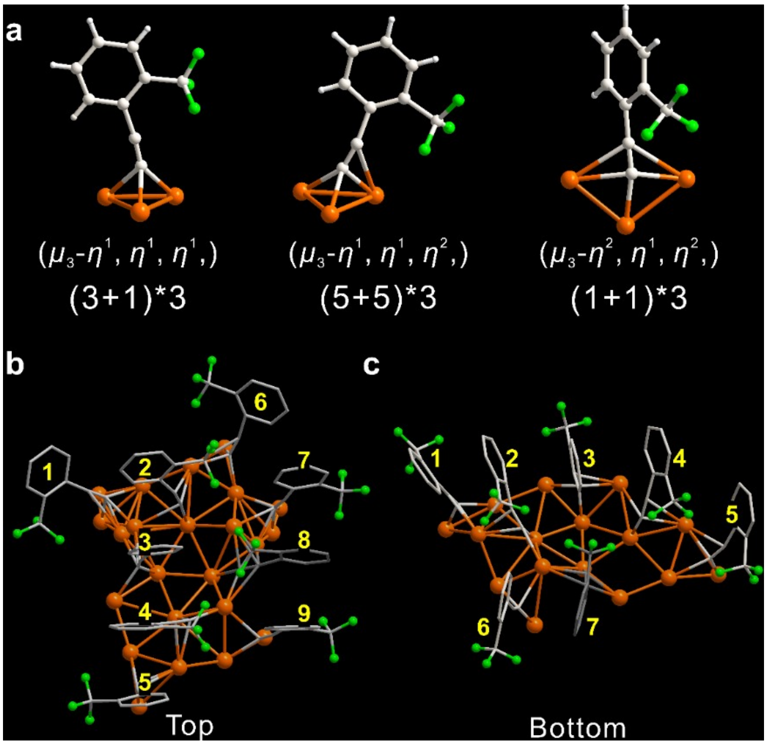


**Figure S4**. (a) Three binding motifs of 2-(trifluoromethyl)phenylacetylene in $\mathbf{Ag_{98}}$, $\mu_3$-$\eta^1$, $\eta^1$, $\eta^1$, $\mu_3$-$\eta^1$, $\eta^1$, $\eta^2$ and $\mu_3$-$\eta^2$, $\eta^1$, $\eta^2$. The number of (b) one leaflet of "three-leaf clover" and (c) one-third of "shield-like" *o*-TPAs in $\mathbf{Ag_{98}}$. Color legend: Ag, orange; C, grey; F, fluorescent green; H, white.

**Table S1.** Angle of inclination of alkynyl ligands at different positions in $\mu_3$-$\eta^1$, $\eta^1$, $\eta^1$ and $\mu_3$-$\eta^1$, $\eta^1$, $\eta^2$ coordination mode relative to the normal to the $Ag_3$ plane. *O*-TPAs of top-8 and bottom-7 adopt $\mu_3$-$\eta^2$, $\eta^1$, $\eta^2$ coordination mode.

| **Top** | | **Bottom** | |
|---|---|---|---|
| O-TPA | Angles of inclination/° | O-TPA | Angles of inclination/° |
| 1 | 17 | 1 | 33 |
| 2 | 51 | 2 | 39 |
| 3 | 35 | 3 | 39 |
| 4 | 34 | 4 | 43 |
| 5 | 35 | 5 | 31 |
| 6 | 14 | 6 | 30 |
| 7 | 17 | 7 | – |
| 8 | – | | |
| 9 | 16 | | |

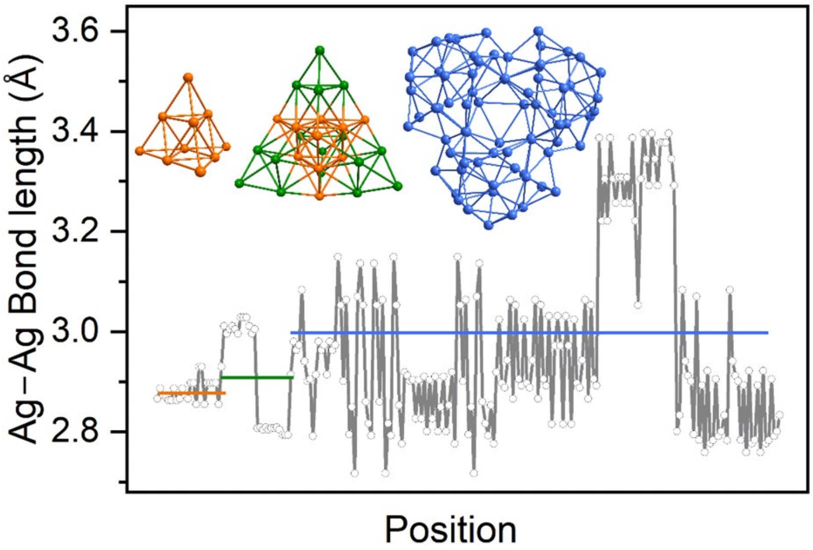


**Figure S5**. Ag–Ag bond length distributions in the kernel of $\mathbf{Ag_{98}}$ nanoclusters: between the center $Ag_{10}$ atoms; between the $Ag_{16}$ shell atoms; between the $Ag_{72}$ core atoms.

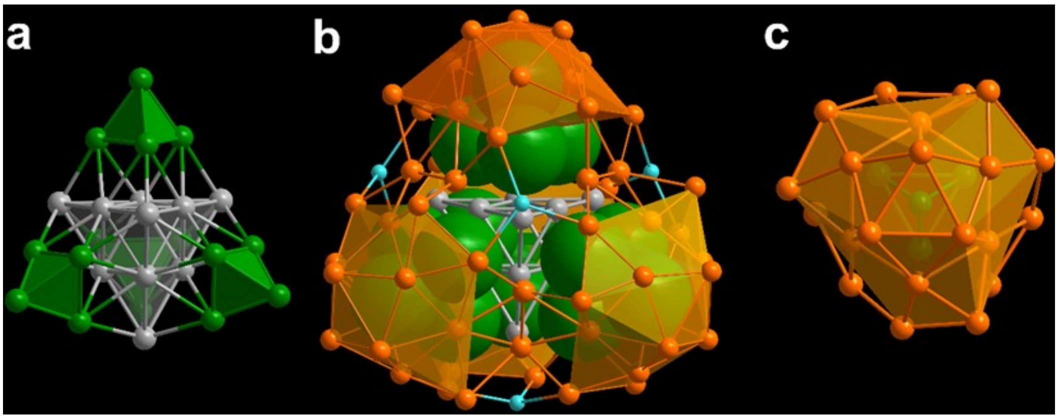


**Figure S6.** (a) $Ag_{10}@Ag_{16}$ core. (b) $Ag_{10}@Ag_{16}@Ag_{72}$ core. (c) Bowl-like $Ag_{18}$ unit encapsulating an $Ag_4$ tetrahedron.

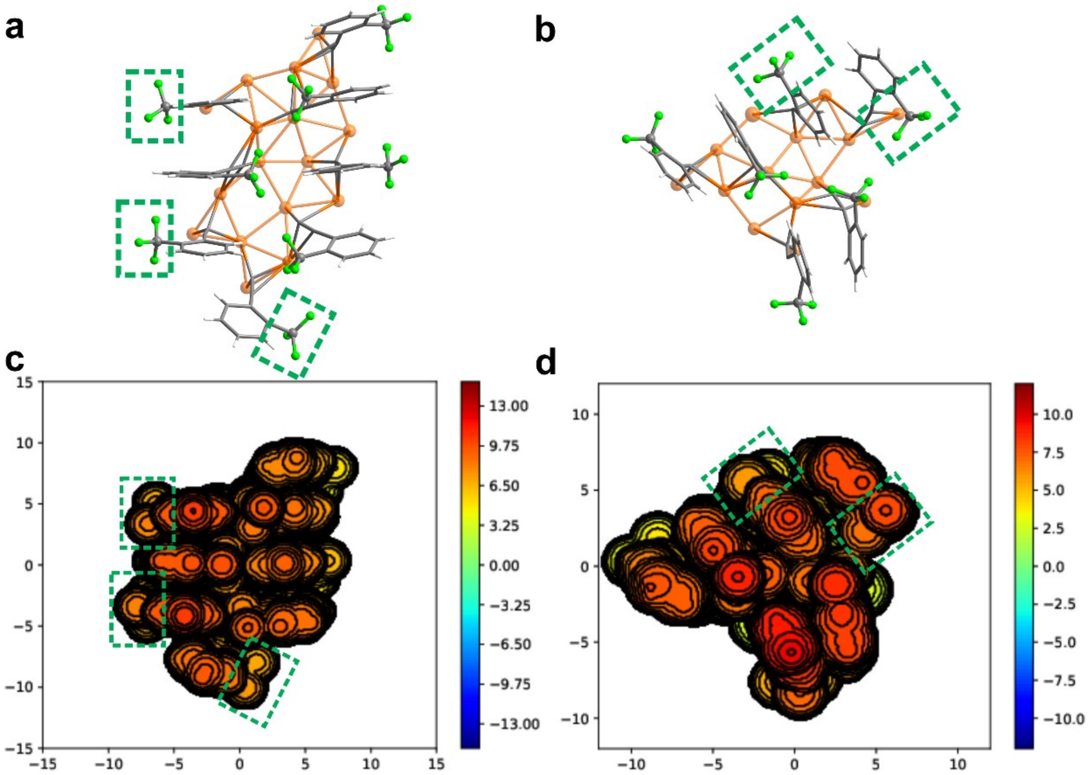


**Figure S7.** Structure and steric maps of (a, c) one leaflet of "three-leaf clover", (b, d) one-third of "shield-like" shape in **$Ag_{98}$**. Some *ortho*-$CF_3$ are marked with green dashed boxes.

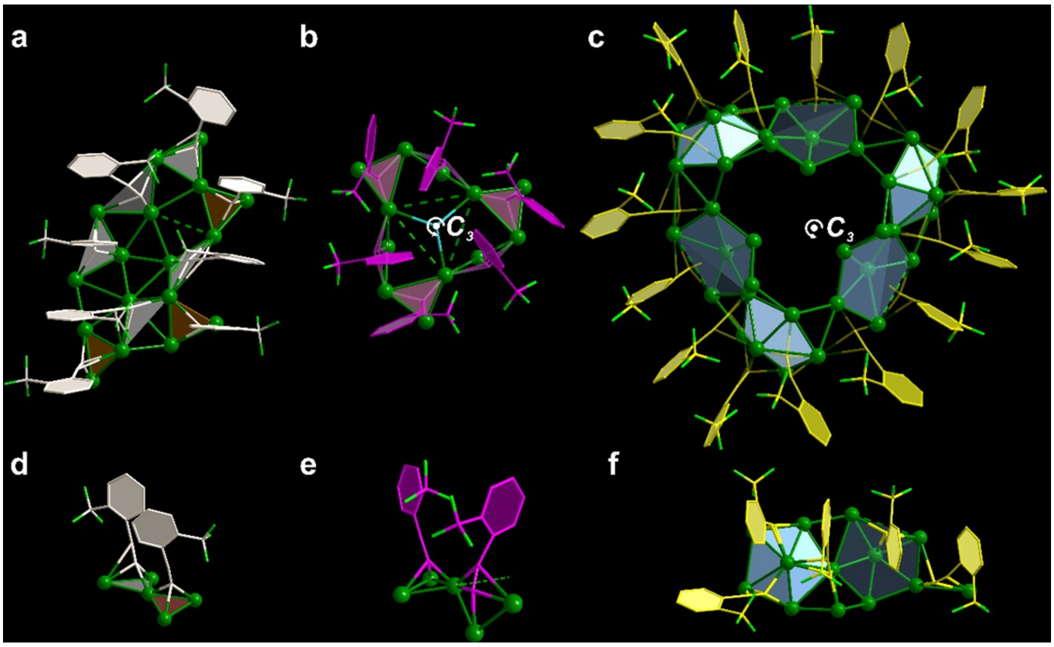


**Figure S8.** Distribution of *o*-TPAs on the "leaflet" (a) and on the "shield" (b, c) of $\mathbf{Ag_{98}}$. (d) Coordination modes of *o*-TPAs on the "leaflet" of $\mathbf{Ag_{98}}$. (e, f). Coordination modes of *o*-TPAs on the "shield" of $\mathbf{Ag_{98}}$. Color labels: green, Ag; gray, purple and yellow, C; fluorescent green, F; blue, Cl; white, H.

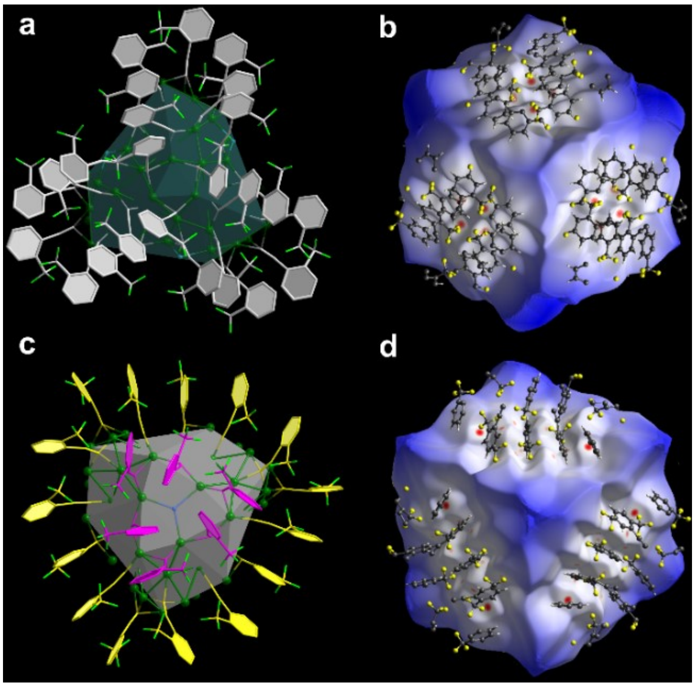


**Figure S9.** Comparison of structures and $d_{\text{norm}}$ surfaces of $\mathbf{Ag_{98}}$. (a) "three-leaf clover" and (c) "shield-like" structure at the top and bottom of $\mathbf{Ag_{98}}$. The $d_{\text{norm}}$ surfaces and adjacent groups on the top (b) and bottom (d) of $\mathbf{Ag_{98}}$.

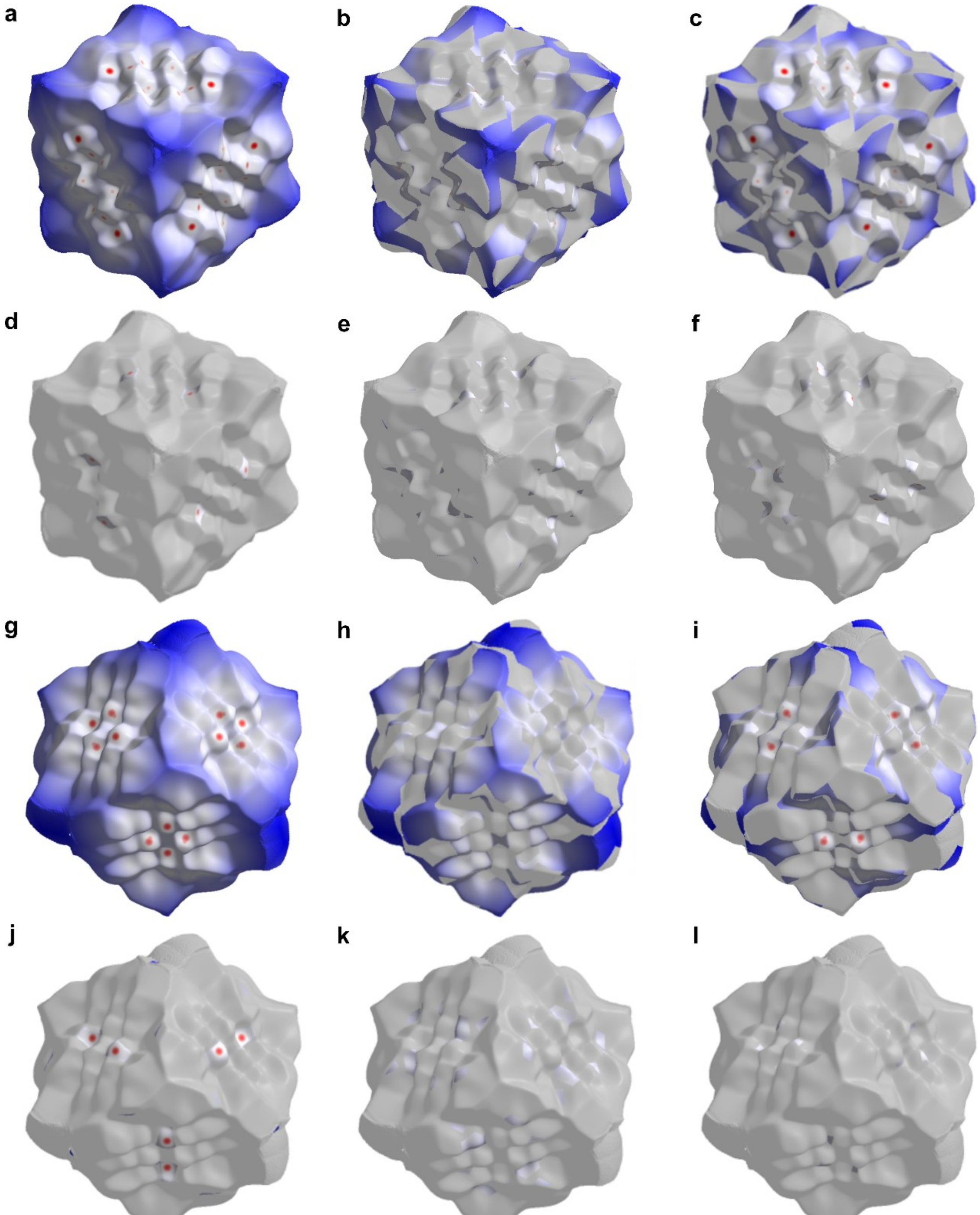


**Figure S10.** (a, g) Hirshfeld surface of **$Ag_{98}$** mapped with $d_{norm}$. Selective highlighting of (b, h) H···H, (c, i) H···F/F···H, (d, j) F···F, (e, k) C···H/H···C and (f, l) C···F contacts on the $d_{norm}$ surface of **$Ag_{98}$**. a-f correspond to the "shield-like" bottom, and g-l correspond to the "three-leaf clover" top.

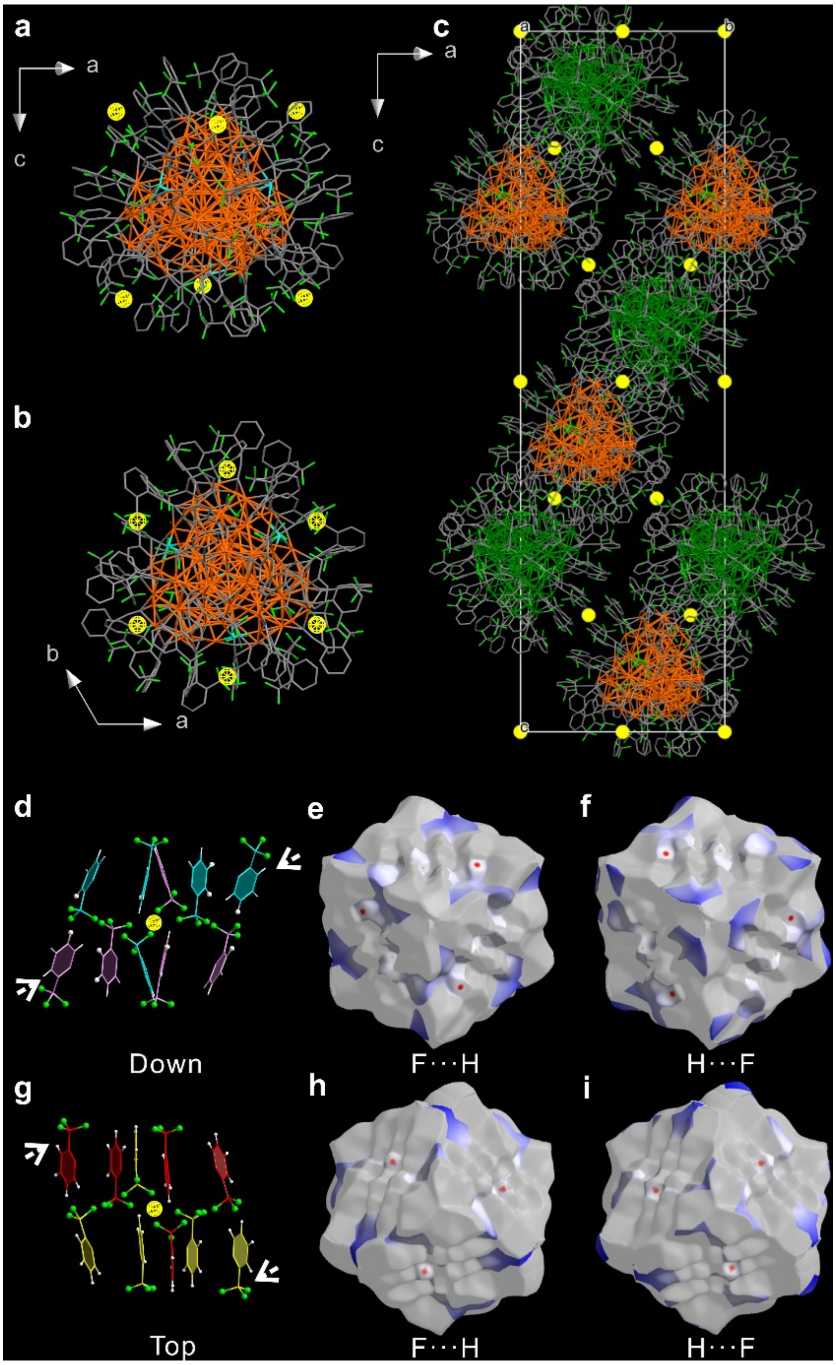


**Figure S11.** (a, b) The location of the inversion center around the $\mathbf{Ag_{98}}$. (c) The location of the inversion center in the unit cell of $\mathbf{Ag_{98}}$. Ligand orientations are distributed centrosymmetrically around the inversion centers of adjacent ligands at the (d) bottom and (g) top between nanoclusters. The inversion center is represented by a yellow ball. The selectively highlighting of F···H or H···F Hirshfeld surfaces at the (e, f) bottom and (h, i) top show the same symmetrical distribution.

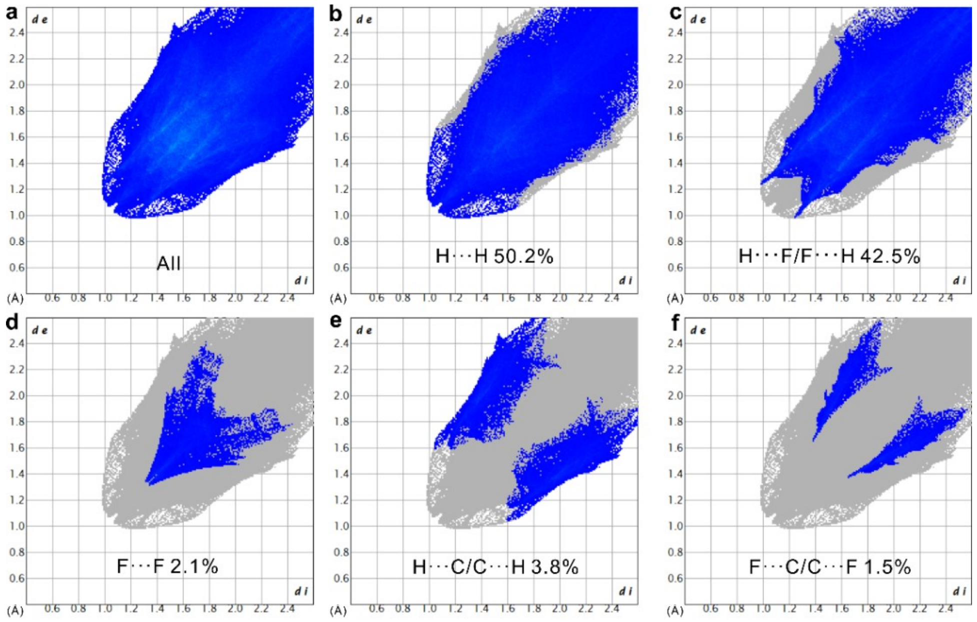


**Figure S12.** (a) The full 2D fingerprint plots for **$Ag_{98}$**. Fingerprinting plots of **$Ag_{98}$** showing the proportion of (b) H···H, (c) H···F/F···H, (d) F···F, (e) C···H/H···C and (f) C···F. The full fingerprint appears beneath each decomposed plot as a grey shadow.

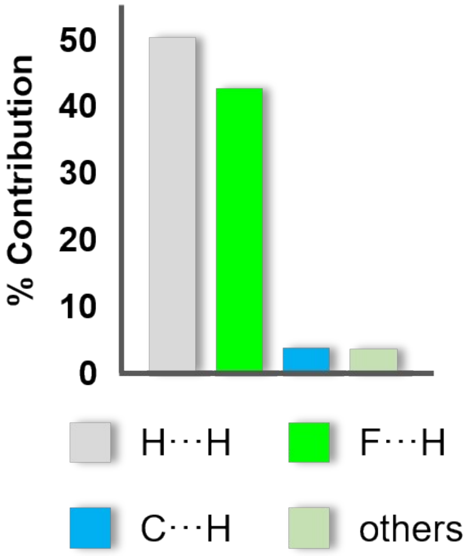


**Figure S13.** Relative contributions to the Hirshfeld surface area for the various close contacts in **$Ag_{98}$**.

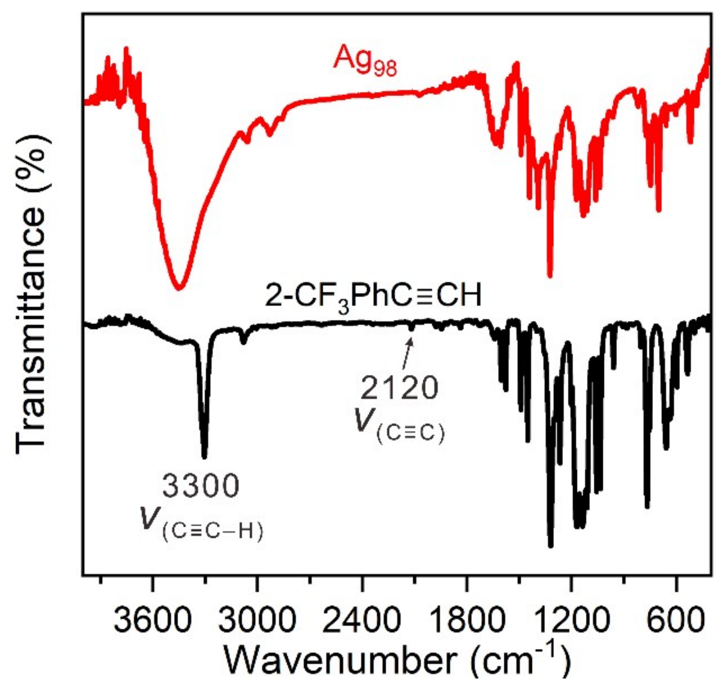


**Figure S14.** FT-IR spectra of **$Ag_{98}$** nanoclusters and 2-$CF_3$PhC≡CH.

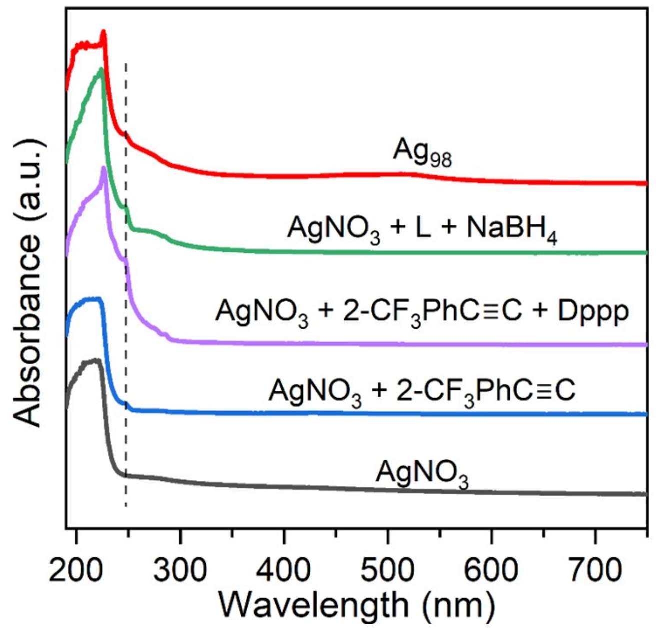


**Figure S15.** UV-vis absorption spectra tracing the formation process of **$Ag_{98}$** nanoclusters. There is always an absorption peak of Ag-alkynyl at 247 nm.

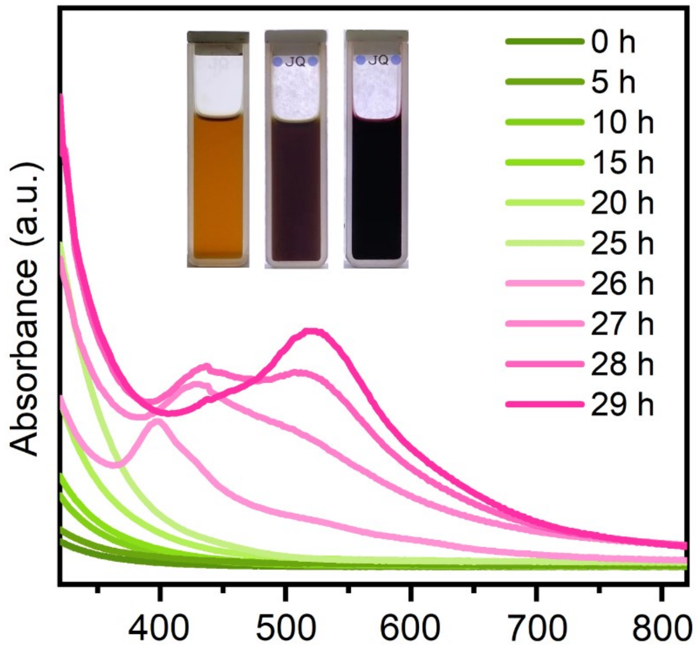


**Figure S16.** UV-vis absorption spectra tracing the formation process of $\mathbf{Ag_{98}}$. Inset: photographs of the reduction process.

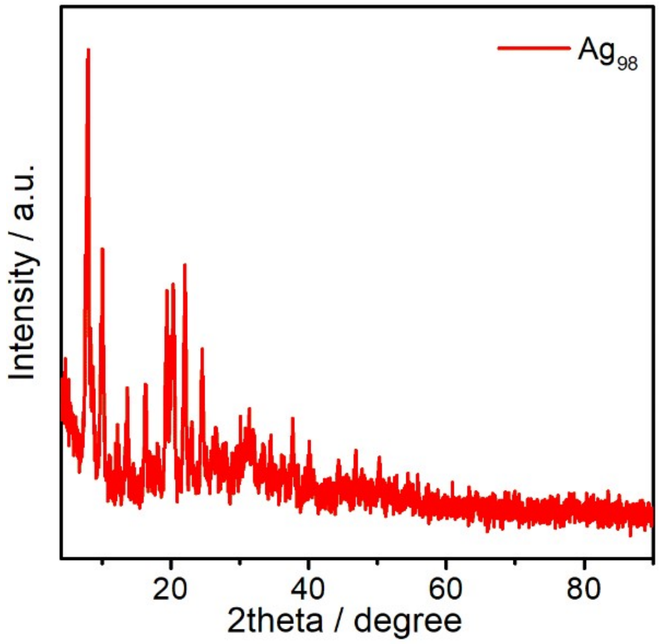


**Figure S17.** The PXRD spectrum of $\mathbf{Ag_{98}}$ nanoclusters.

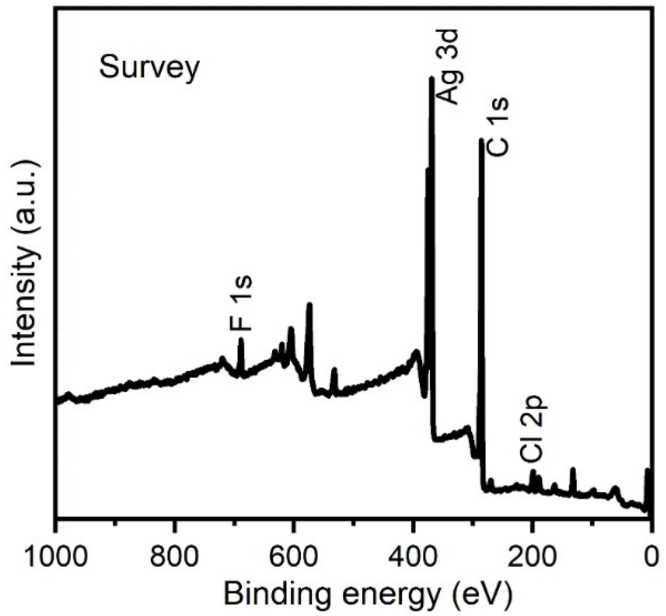


**Figure S18.** A Survey scan of XPS spectrum of **$Ag_{98}$** nanoclusters.

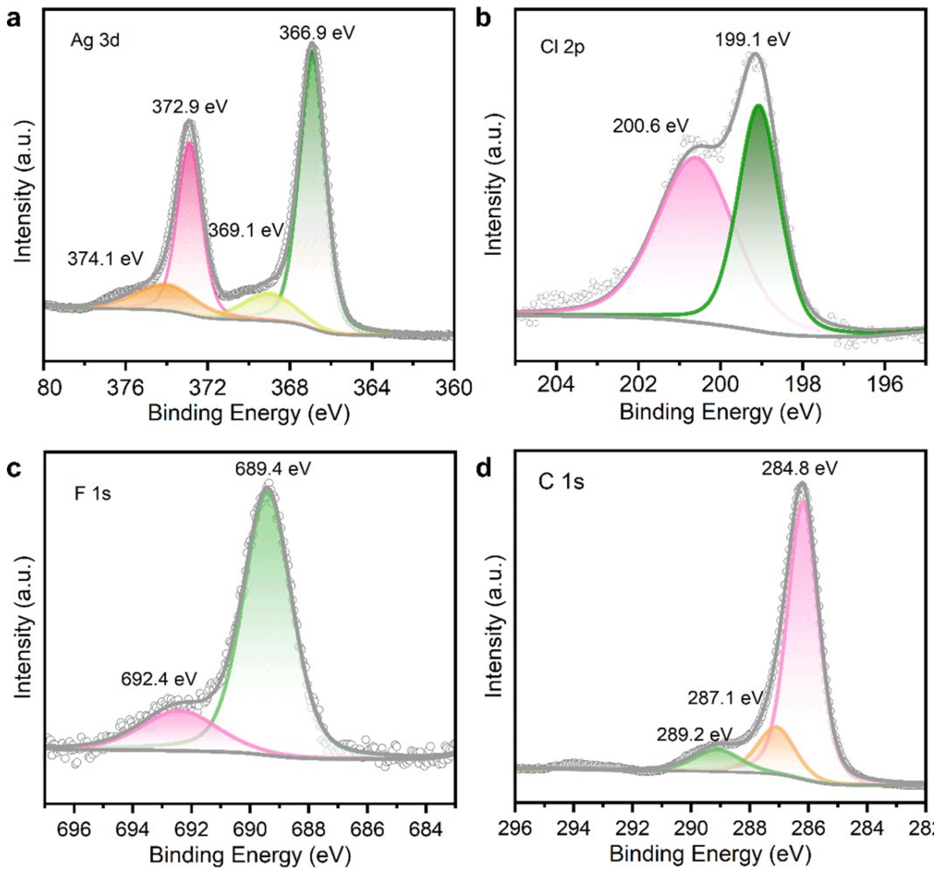


**Figure S19.** High-resolution XPS spectra of Ag 3d, Cl 2p, F 1s and C 1s for **$Ag_{98}$** nanoclusters. XPS spectra in the F 1s, two peaks are observed at the binding energies of 692.4 and 689.4 eV.

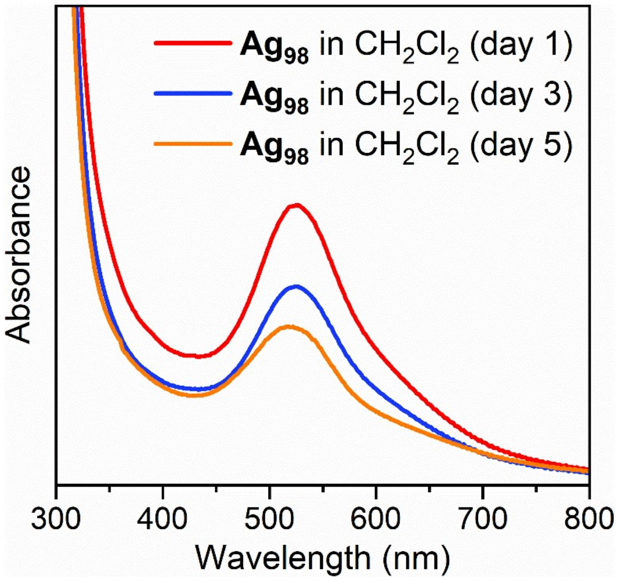


**Figure S20.** UV-Vis absorption spectra of as-synthesized $\mathbf{Ag_{98}}$ solution and that after stored at ambient conditions for 3 and 5 days. $\mathbf{Ag_{98}}$ is fairly stable. The as-prepared solution can be stored at ambient conditions for at least 5 days without change.

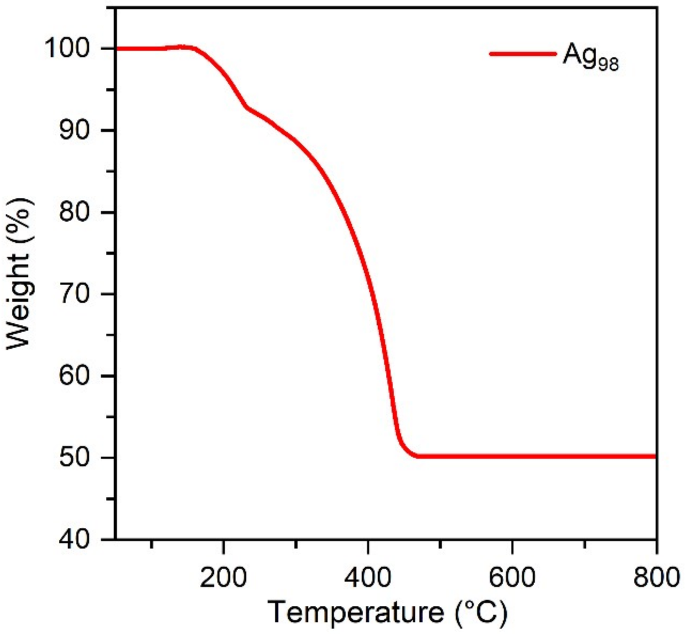


**Figure S21.** TGA curve of $\mathbf{Ag_{98}}$ nanoclusters.

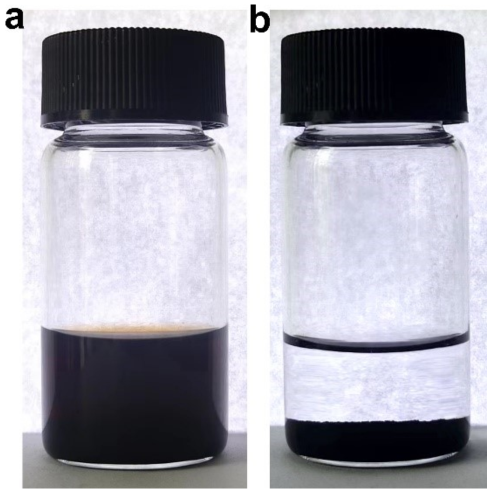


**Figure S22.** Immobilization of **$Ag_{98}$** on carbon black. Solution before (a) and after (b) addition of carbon black. After adding carbon black, the solution gradually becomes colorless.

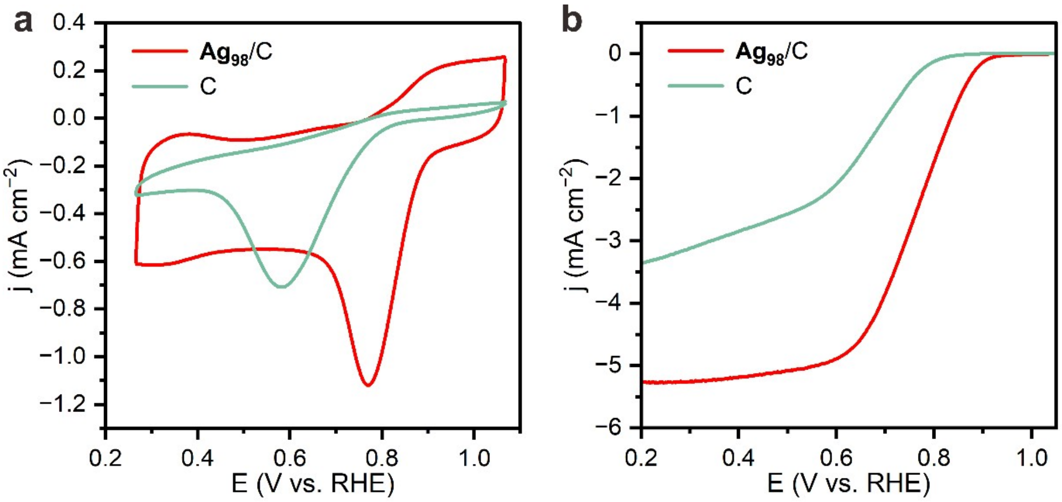


**Figure S23.** (a) CV and (b) LSV curves of the **$Ag_{98}$**/C and carbon black.

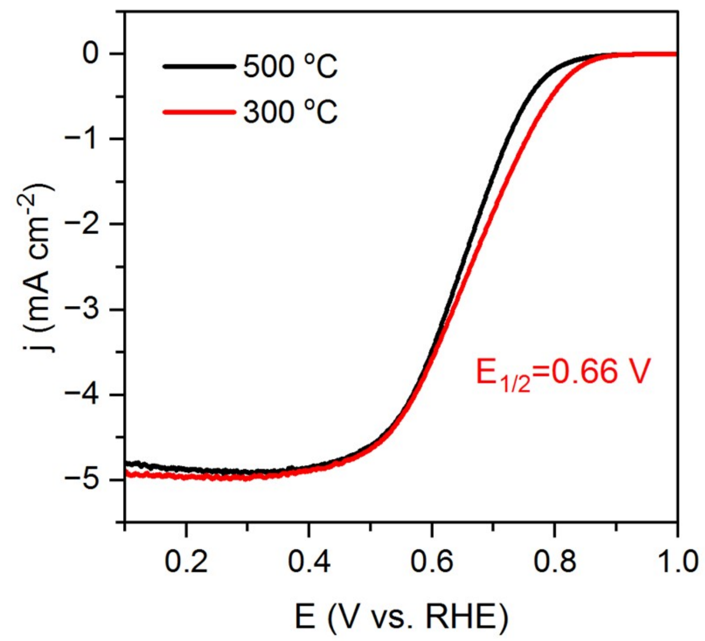


**Figure S24.** ORR LSV curves of $\mathbf{Ag_{98}}$/C catalysts calcined at 300 ℃ and 500 ℃.

**Table S2** Comparison of the ORR catalytic performance of $\mathbf{Ag_{98}}$/C in this work with reported similar materials.

| sample | $E_{onset}$ (V) | $E_{1/2}$ (V) | $-j_L$ (mA cm$^{-2}$) | Tafel slope (mV dec$^{-1}$) | Electrolyte | Reference |
|---|---|---|---|---|---|---|
| $\mathbf{Ag_{98}}$/C | 0.91 | 0.76 | 5.2 | 56.9 | 0.1 M KOH | This work |
| $Ag_{213}$/C | 0.89 | 0.72 | 4.8 | — | 0.1 M KOH | [4] |
| Ag NPs | 0.88 | 0.76 | 3.5 | — | 0.1 M KOH | [5] |
| $Au_7Ag_6$-0/C | 0.79 | 0.67 | 4.5 | — | 0.1 M KOH | [6] |
| $[Ag_xAu_{25-x}(SC_6H_{11})_{18}]^-$ | 0.80 | 0.66 | 4.0 | — | 0.1 M KOH | [7] |
| $Au_{52}$-PAP | 0.90 | 0.69 | 5.5 | — | 0.1 M KOH | [8] |
| $Ag_{29}(PPh_3)_4(BDT)_{12}$/C | 0.78 | 0.67 | 3.8 | — | 0.1 M KOH | [9] |
| AgNCs@GO | 0.95 | 0.92 | 5.8 | 45.0 | 0.1 M KOH | [10] |
| $Fe_{28}$ | 0.93 | 0.84 | 5.6 | 106.6 | 0.1 M KOH | [11] |
| $Pt_{17}$/CB | ~0.76 | ~0.62 | 5.0 | — | 0.1 M $HClO_4$ | [12] |
| AuPC-1 | 0.95 | 0.82 | 3.7 | — | 0.1 M KOH | [13] |

**Table S3 Crystal data and structure refinement for $Ag_{98}$.**

| Identification code | **$Ag_{98}$** |
|---|---|
| Empirical formula | $C_{432}H_{192}Ag_{98}Cl_4F_{144}$ |
| Formula weight | 18830.90 |
| Temperature/K | 150.00(10) |
| Crystal system | trigonal |
| Space group | $R\bar{3}$ |
| $a$/Å | 32.4731(4) |
| $b$/Å | 32.4731(4) |
| $c$/Å | 96.4532(11) |
| $\alpha$/° | 90 |
| $\beta$/° | 90 |
| $\gamma$/° | 120 |
| Volume/Å$^3$ | 88084(2) |
| Z | 6 |
| $\rho_{calc}$g/cm$^3$ | 2.130 |
| $\mu$/mm$^{-1}$ | 26.424 |
| F(000) | 52524.0 |
| Crystal size/mm$^3$ | 0.271 × 0.271 × 0.268 |
| Radiation | Cu Kα (λ = 1.54184) |
| 2Θ range for data collection/° | 6.098 to 153.082 |
| Index ranges | $-35 \leq h \leq 30$, $-20 \leq k \leq 40$, $-121 \leq l \leq 118$ |
| Reflections collected | 98169 |
| Independent reflections | 39373 [$R_{int}$ = 0.0609, $R_{sigma}$ = 0.0735] |
| Data/restraints/parameters | 39373/1876/1908 |
| Goodness-of-fit on F$^2$ | 0.949 |
| Final $R$ indexes [I>=2σ (I)] | $R_1$ = 0.0820, $wR_2$ = 0.2515 |
| Final $R$ indexes [all data] | $R_1$ = 0.1313, $wR_2$ = 0.2947 |
| Largest diff. peak/hole / e Å$^{-3}$ | 1.88/-1.86 |

**References**

(1) S. Chiodo; N. Russo and E. SiCilia, *J. Chem. Phys.* 2006, 125, 104107.

(2) T. Lu, F. Chen, *J. Comput. Chem.*, 2012, 33, 580–592.

(3) W. Humphrey, A. Dalke, K. Schulten, *J. Mol. Graph.*, 1996, 14, 33–38.

(4) C.-G. Shi, J.-H. Jia, Y. Jia, G. Li and M.-L. Tong, *CCS Chem.*, 2023, 5, 1154–1162.

(5) X. Yang, L. Gan, C. Zhu, B. Lou, L. Han, J. Wang and E. Wang, *Chem. Commun.*, 2014, 50, 234–236.

(6) M, Zhou, K, Li, Y, Pei, S, Jin, and M. Zhu *J. Phys. Chem. Lett.* 2023, 14, 11715−11724.

(7) R. Jin, S. Zhao, C. Liu, M. Zhou, G. Panapitiya, Y. Xing, N. L. Rosi, J. P. Lewisd and R. Jin, *Nanoscale*, 2017, 9, 19183−19190.

(8) S. Zhuang, D. Chen, W.-P. Ng, D. Liu, L.-J. Liu, M.-Y. Sun, T. Nawaz, X. Wu, Y. Zhang, Z. Li, Y.-L. Huang, J. Yang, J. Yang and J. He, *JACS Au*, 2022, 2, 2617–2626.

(9) X. Zou, S. He, X. Kang, S. Chen, H. Yu, S. Jin, D. Astruc and M. Zhu, *Chem. Sci.*, 2021, 12, 3660–3667.

(10) Z.-D. Wang, Y. Han, W.-Y. Sun, P. Peng and S.-Q. Zang, *Inorg. Chem. Front.*, 2024, DOI: 10.1039/D4QI01335B.

(11) J.-Q. Lv, Z.-L. Lang, J.-Q. Fu+ , Q., R. Liu, H.-Y. Zang, Y.-G. Li,

D.-D. Ye, and C. Streb, *Angew. Chem. Int. Ed.*, 2022, 61, e202202650.

(12) T. Kawawaki, Y. Mitomi, N. Nishi, R. Kurosaki, K. Oiwa, T. Tanaka, H. Hirase, S. Miyajima, Y. Niihori, D. J. Osborn, T. Koitaya, G. F. Metha, T. Yokoyama, K. Iida and Y. Negishi, *Nanoscale*, 2023, 15, 7272–7279.

(13) D.-D. Qin a, Y. Tang, G. Ma, L. Qin, C.-L. Tao, X. Zhang, Z. Tang, *Int. J. Hydrogen Energy*, 2021, 46, 25771–25781.
